# Crystal structure of 4-(4b,8a-di­hydro-9*H*-pyrido[3,4-*b*]indol-1-yl)-7-methyl-2*H*-chromen-2-one

**DOI:** 10.1107/S2056989016019769

**Published:** 2017-01-01

**Authors:** S. Samundeeswari, Manohar V. Kulkarni, G. N. Anil Kumar

**Affiliations:** a Department of Chemistry, Karnatak University, Dharwad, India; b Department of Physics, M. S. Ramaiah Institute of Technology, Bangalore, India

**Keywords:** crystal structure, coumarins, β-carboline, norharman, hydrogen bonding, π-π inter­actions

## Abstract

The title compound, C_21_H_14_N_2_O_2_, was prepared by Pictet–Spengler cyclization of tryptamine and 4-formyl coumarin. In the mol­ecule, the dihedral angle between the mean planes of the coumarin and β-carboline ring systems is 63.8 (2)°. In the crystal, mol­ecules are linked *via* N—H⋯N hydrogen bonds, forming chains along the *b*-axis direction. Within the chains, there are a number of offset π–π inter­actions present [shortest inter­centroid distance = 3.457 (2) Å].

## Abstract 2

In the title compound, the dihedral angle between the mean planes of the coumarin and β-carboline ring systems is 63.8 (2)°. In the crystal, mol­ecules are linked *via* N—H⋯N hydrogen bonds, forming chains along the [010] direction.

## Chemical context   

Naturally occurring coumarins (Murry, 2002 ▸) and their deriv­atives have a vast number of applications in different areas. They are precursor reagents for synthetic anti-coagulants (Bairagi *et al.*, 2012 ▸), the most notable being warfarin (Holbrook *et al.*, 2005 ▸). Coumarin dyes are also widely used in blue–green organic dyes (Schafer, 1990 ▸; Duarte & Hillman, 1990 ▸; Duarte, 2003 ▸) and in OLED emitters (Duarte *et al.*, 2005 ▸). Norharman is a β-carboline alkaloid which has the basic structural unit for a wide range of naturally occurring compounds, and is found in plants, animals and humans (Fekkes *et al.*, 1992 ▸). They are used widely as neurotoxins to Parkinson’s disease (Kuhn *et al.*, 1996 ▸) and as mediators in the mutagenesis of DNA in the presence of another mol­ecule (Mori *et al.*, 1996 ▸). Given the ongoing research into the biological functions of norharman and the many related β-carboline derivatives, a single-crystal X-ray structure of norharman would be of use in theoretical modelling and related structural work. Norharman exhibits a one-dimensional herringbone motif (Thatcher & Douthwaite, 2011 ▸). Due to their extensive natural occurrence and common biological origin, there are no reports on compounds which contain these two systems in a single mol­ecule. It was hence thought of considerable biological inter­est to synthesize new mol­ecules which contain both β-carboline and coumarin ring systems.
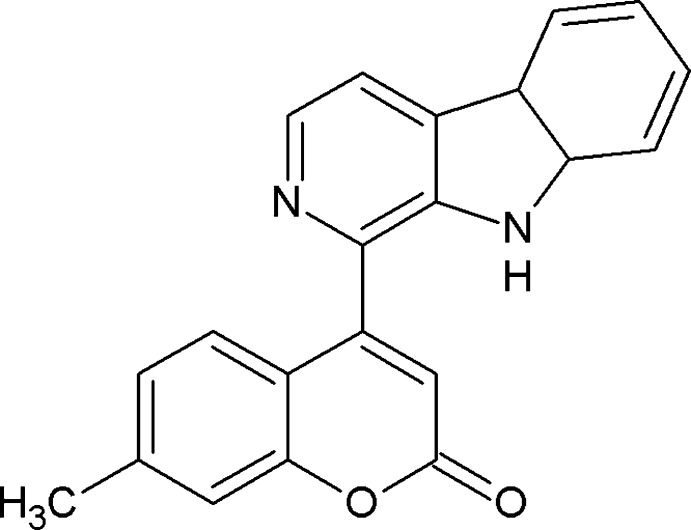


## Structural commentary   

**Figure 1:**
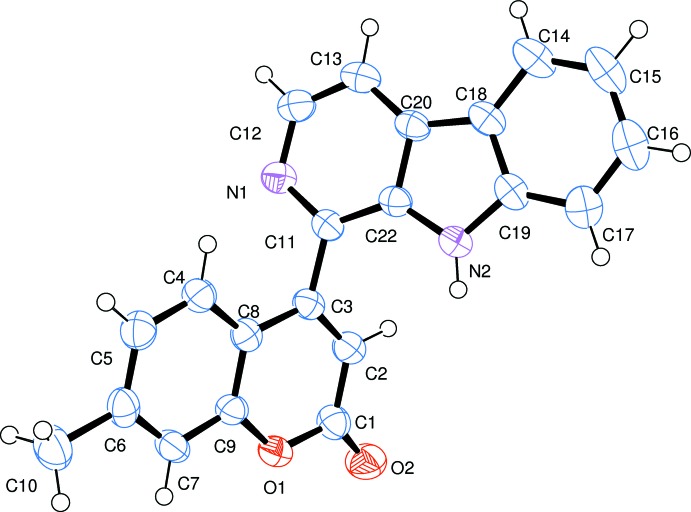
The mol­ecular structure of the title compound, with the atom labelling and displacement ellipsoids drawn at the 50% probability level.

The mol­ecular structure of the title compound is shown in Fig. 1 ▸. The coumarin (r.m.s. deviation = 0.019 Å) and β-carboline (r.m.s. deviation = 0.034 Å) ring systems exhibit an *s-trans* arrangement across the bridging C7—C6 bond; their mean planes are inclined to one another by 63.8 (2)°.

## Supra­molecular features   

**Figure 2:**
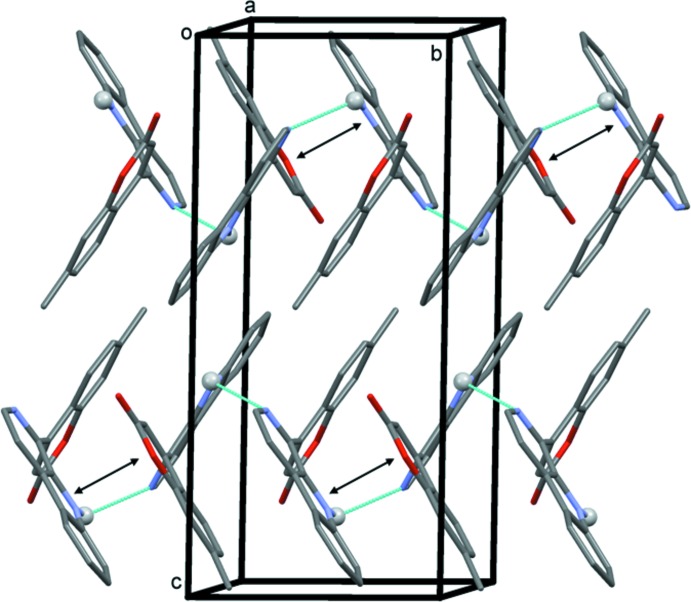
A view along the *a* axis of the crystal packing of the title compound. The N—H⋯N hydrogen bonds are shown as dashed lines (see Table 1 ▸), and the shortest offset π–π inter­actions by a double-headed arrow. For clarity, only H atom H2 (grey ball) has been included.

In the crystal, mol­ecules are linked *via* N—H⋯N hydrogen bonds, forming chains along [010]; see Table 1 ▸ and Fig. 2 ▸. Within the chains there are a number of offset π–π inter­actions present; the shortest inter­centroid distance of 3.457 (2) Å, involves rings N2/C18–C20/C22 of the β-carboline ring system and O1/C1–C3/C8/C9 of the coumarin system.

## Database survey   

A search of the Cambridge Structural Database (CSD, Version 5.37, last update May 2016; Groom *et al.*, 2016 ▸) using 4,7-dimethyl-2*H*-chromen-2-one as the main skeleton revealed the presence of 66 structures. However, only six of these structures contain the 7-methyl-4-phenyl-2*H*-chromen-2-one nucleus (refcodes: BUFQUQ, FINNEX, GUFTUY, IFUMED, LENYIO, DUVVIB). There were no structures reported for a search of 7-methyl-4-(pyridin-2-yl)-2*H*-chromen-2-one skeleton.

## Synthesis and crystallization   

Acetic acid (10 ml) was added drop wise, at 273 K, to a mixture of tryptamine (1 eq) and 4-formyl coumarin (1 eq). The reaction mixture was stirred at room temperature for *ca* 12 h. After completion of the reaction, the solid that separated was filtered, washed several times with water and dried (yield >70%) to give the inter­mediate. This inter­mediate compound (1 eq) was taken in 10 ml of dry chloro­form and 2,3-di­chloro-5,6-di­cyano-1,4-benzo­quinone (2 eq) was added at inter­vals of 5 min in cold conditions, 273 K. Stirring was continued for *ca* 10 h. The reaction mixture was then quenched using aqueous sodium bicarbonate and extracted with chloro­form. The organic layer was washed 2–3 times with sodium bicarbonate, water and brine solution, dried using sodium sulfate, and concentrated to afford the crude title product. It was purified by flash chromatography using 230–400 mesh silica-gels (35% ethyl acetate in hexane mixture; yield 75%). The solid obtained was recrystallized from dichloromethane, giving colourless block-like crystals of the title compound on slow evaporation of the solvent

## Refinement   

Crystal data, data collection and structure refinement details are summarized in Table 2 ▸. H atoms were positioned geometrically, with N—H = 0.86 Å and C—H = 0.93–0.96 Å, and constrained to ride on their parent atoms with *U*_iso_(H) = 1.5*U*_eq_(C-meth­yl) and 1.2*U*_eq_(C,N) for other H atoms.

## Supplementary Material

Crystal structure: contains datablock(s) global, I. DOI: 10.1107/S2056989016019769/su5339sup1.cif

Structure factors: contains datablock(s) I. DOI: 10.1107/S2056989016019769/su5339Isup2.hkl

Supporting information file. DOI: 10.1107/S2056989016019769/su5339Isup3.cml

CCDC reference: 1522101

Additional supporting information: crystallographic information; 3D view; checkCIF report

## Figures and Tables

**Table 1:** Hydrogen-bond geometry (Å, °)

*D*—H⋯*A*	*D*—H	H⋯*A*	*D*⋯*A*	*D*—H⋯*A*
N2—H2⋯N1^i^	0.86	2.47	2.994 (3)	120

Symmetry code: (i) 
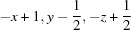
.

**Table 2:** Experimental details

Crystal data	
Chemical formula	C_21_H_14_N_2_O_2_
*M*_r_	326.34
Crystal system, space group	Monoclinic, *P*2_1_/*c*
Temperature (K)	296
*a*, *b*, *c* (Å)	10.6784 (8), 8.0954 (6), 17.9032 (14)
β (°)	98.105 (5)
*V* (Å^3^)	1532.2 (2)
*Z*	4
Radiation type	Mo *K*α
μ (mm^−1^)	0.09
Crystal size (mm)	0.20 × 0.15 × 0.10

Data collection	
Diffractometer	Bruker SMART CCD area-detector
Absorption correction	Multi-scan (*SADABS*; Bruker, 2012 ▸)
*T*_min_, *T*_max_	0.941, 0.971
No. of measured, independent and observed [*I* > 2σ(*I*)] reflections	11601, 2848, 1446
*R*_int_	0.059
(sin θ/λ)_max_ (Å^−1^)	0.606

Refinement	
*R*[*F* ^2^ > 2σ(*F* ^2^)], *wR*(*F* ^2^), *S*	0.060, 0.166, 0.94
No. of reflections	2848
No. of parameters	227
H-atom treatment	H-atom parameters constrained
Δρ_max_, Δρ_min_ (e Å^−3^)	0.21, −0.27

Computer programs: *SMART* and *SAINT* (Bruker, 2012 ▸), *SHELXS97* (Sheldrick, 2008 ▸), *SHELXL2014* (Sheldrick, 2015 ▸), *ORTEP-3 for Windows* and *WinGX* (Farrugia, 2012 ▸), *Mercury* (Macrae *et al.*, 2008 ▸) and *PLATON* (Spek, 2009 ▸).

## supplementary crystallographic information

### Crystal data

**Table:**

C_21_H_14_N_2_O_2_	*F*(000) = 680
*M**_r_* = 326.34	*D*_x_ = 1.415 Mg m^−^^3^
Monoclinic, *P*2_1_/*c*	Mo *K*α radiation, λ = 0.71073 Å
Hall symbol: -P 2ybc	Cell parameters from 1990 reflections
*a* = 10.6784 (8) Å	θ = 3.3–26.4°
*b* = 8.0954 (6) Å	µ = 0.09 mm^−^^1^
*c* = 17.9032 (14) Å	*T* = 296 K
β = 98.105 (5)°	Block, colourless
*V* = 1532.2 (2) Å^3^	0.20 × 0.15 × 0.10 mm
*Z* = 4	

### Data collection

**Table:**

Bruker SMART CCD area-detector diffractometer	2848 independent reflections
Radiation source: fine-focus sealed tube	1446 reflections with *I* > 2σ(*I*)
Graphite monochromator	*R*_int_ = 0.059
ω and φ scans	θ_max_ = 25.5°, θ_min_ = 1.9°
Absorption correction: multi-scan (SADABS; Bruker, 2012)	*h* = −12→11
*T*_min_ = 0.941, *T*_max_ = 0.971	*k* = −9→9
11601 measured reflections	*l* = −21→21

### Refinement

**Table:**

Refinement on *F*^2^	Primary atom site location: structure-invariant direct methods
Least-squares matrix: full	Secondary atom site location: difference Fourier map
*R*[*F*^2^ > 2σ(*F*^2^)] = 0.060	Hydrogen site location: inferred from neighbouring sites
*wR*(*F*^2^) = 0.166	H-atom parameters constrained
*S* = 0.94	*w* = 1/[σ^2^(*F*_o_^2^) + (0.0799*P*)^2^] where *P* = (*F*_o_^2^ + 2*F*_c_^2^)/3
2848 reflections	(Δ/σ)_max_ < 0.001
227 parameters	Δρ_max_ = 0.21 e Å^−^^3^
0 restraints	Δρ_min_ = −0.27 e Å^−^^3^

### Special details

**Table:**

Geometry. All esds (except the esd in the dihedral angle between two l.s. planes) are estimated using the full covariance matrix. The cell esds are taken into account individually in the estimation of esds in distances, angles and torsion angles; correlations between esds in cell parameters are only used when they are defined by crystal symmetry. An approximate (isotropic) treatment of cell esds is used for estimating esds involving l.s. planes.

### Fractional atomic coordinates and isotropic or equivalent isotropic displacement parameters (Å^2^)

**Table:**

	*x*	*y*	*z*	*U*_iso_*/*U*_eq_	
O1	0.85044 (16)	0.1787 (2)	0.24656 (10)	0.0445 (6)	
O2	0.87191 (18)	0.3169 (3)	0.35363 (12)	0.0602 (7)	
N1	0.3818 (2)	0.2835 (3)	0.17822 (12)	0.0413 (7)	
N2	0.4360 (2)	0.0447 (3)	0.35591 (12)	0.0407 (7)	
H2	0.5149	0.0259	0.3707	0.049*	
C1	0.7990 (3)	0.2565 (4)	0.30347 (17)	0.0429 (8)	
C2	0.6635 (2)	0.2570 (3)	0.29733 (15)	0.0427 (8)	
H2A	0.6264	0.3055	0.3359	0.051*	
C3	0.5878 (2)	0.1912 (3)	0.23877 (15)	0.0341 (7)	
C4	0.5774 (3)	0.0283 (4)	0.11849 (15)	0.0401 (8)	
H4	0.4895	0.0273	0.1126	0.048*	
C5	0.6389 (3)	−0.0500 (4)	0.06608 (15)	0.0459 (8)	
H5	0.5921	−0.1047	0.0258	0.055*	
C6	0.7705 (3)	−0.0486 (4)	0.07241 (15)	0.0427 (8)	
C7	0.8384 (3)	0.0291 (4)	0.13369 (15)	0.0431 (8)	
H7	0.9263	0.0302	0.1395	0.052*	
C8	0.6443 (2)	0.1088 (3)	0.18005 (15)	0.0353 (7)	
C9	0.7754 (2)	0.1051 (4)	0.18637 (15)	0.0370 (7)	
C10	0.8380 (3)	−0.1277 (4)	0.01293 (17)	0.0635 (10)	
H10A	0.924	−0.1509	0.0339	0.095*	
H10B	0.8369	−0.0537	−0.0291	0.095*	
H10C	0.7961	−0.2287	−0.0038	0.095*	
C11	0.4475 (2)	0.2009 (4)	0.23601 (15)	0.0356 (7)	
C12	0.2541 (3)	0.2974 (4)	0.17666 (16)	0.0464 (8)	
H12	0.2093	0.3581	0.1376	0.056*	
C13	0.1874 (3)	0.2280 (4)	0.22862 (17)	0.0449 (8)	
H13	0.1	0.2388	0.2242	0.054*	
C14	0.1087 (3)	0.0115 (4)	0.37782 (19)	0.0542 (9)	
H14	0.0321	0.0482	0.3519	0.065*	
C15	0.1115 (3)	−0.0820 (4)	0.44170 (19)	0.0595 (10)	
H15	0.0361	−0.1107	0.4587	0.071*	
C16	0.2255 (3)	−0.1345 (4)	0.48153 (18)	0.0589 (9)	
H16	0.2248	−0.1961	0.5253	0.071*	
C17	0.3403 (3)	−0.0978 (4)	0.45785 (16)	0.0489 (9)	
H17	0.4166	−0.1329	0.4847	0.059*	
C18	0.2223 (3)	0.0506 (4)	0.35234 (15)	0.0411 (8)	
C19	0.3358 (3)	−0.0063 (4)	0.39225 (15)	0.0403 (7)	
C20	0.2544 (2)	0.1410 (3)	0.28810 (16)	0.0370 (7)	
C22	0.3865 (2)	0.1313 (3)	0.29183 (15)	0.0342 (7)	

### Atomic displacement parameters (Å^2^)

**Table:**

	*U*^11^	*U*^22^	*U*^33^	*U*^12^	*U*^13^	*U*^23^
O1	0.0319 (11)	0.0588 (15)	0.0433 (12)	−0.0005 (10)	0.0069 (9)	−0.0029 (11)
O2	0.0450 (13)	0.0760 (18)	0.0575 (14)	−0.0050 (12)	−0.0001 (11)	−0.0134 (13)
N1	0.0356 (14)	0.0428 (17)	0.0454 (15)	0.0022 (12)	0.0056 (11)	0.0007 (13)
N2	0.0321 (13)	0.0503 (18)	0.0406 (14)	0.0004 (12)	0.0083 (11)	0.0003 (13)
C1	0.0423 (18)	0.044 (2)	0.0434 (18)	−0.0038 (16)	0.0090 (15)	−0.0018 (16)
C2	0.0376 (17)	0.049 (2)	0.0419 (18)	0.0018 (15)	0.0083 (14)	−0.0027 (16)
C3	0.0332 (15)	0.0306 (19)	0.0397 (16)	0.0001 (13)	0.0086 (13)	0.0041 (14)
C4	0.0372 (16)	0.043 (2)	0.0409 (17)	−0.0026 (15)	0.0071 (13)	0.0029 (15)
C5	0.048 (2)	0.048 (2)	0.0419 (18)	−0.0040 (16)	0.0066 (15)	0.0008 (16)
C6	0.055 (2)	0.037 (2)	0.0376 (17)	0.0076 (16)	0.0135 (15)	0.0056 (15)
C7	0.0359 (16)	0.049 (2)	0.0468 (18)	0.0071 (15)	0.0150 (14)	0.0058 (16)
C8	0.0385 (17)	0.0310 (19)	0.0373 (16)	−0.0013 (14)	0.0086 (13)	0.0032 (14)
C9	0.0311 (16)	0.041 (2)	0.0393 (17)	−0.0008 (14)	0.0060 (13)	0.0051 (15)
C10	0.070 (2)	0.071 (3)	0.054 (2)	0.0156 (19)	0.0223 (17)	−0.0037 (19)
C11	0.0333 (16)	0.036 (2)	0.0382 (16)	−0.0008 (14)	0.0086 (13)	−0.0025 (14)
C12	0.0383 (18)	0.050 (2)	0.0500 (19)	0.0041 (16)	0.0011 (15)	−0.0005 (16)
C13	0.0320 (16)	0.045 (2)	0.058 (2)	0.0013 (15)	0.0074 (15)	−0.0065 (17)
C14	0.0461 (19)	0.048 (2)	0.074 (2)	0.0008 (17)	0.0276 (17)	−0.0005 (19)
C15	0.064 (2)	0.051 (2)	0.073 (2)	−0.0048 (19)	0.0414 (19)	−0.001 (2)
C16	0.078 (2)	0.049 (2)	0.056 (2)	−0.006 (2)	0.0313 (19)	0.0005 (18)
C17	0.059 (2)	0.041 (2)	0.0487 (19)	0.0011 (17)	0.0127 (16)	0.0001 (16)
C18	0.0375 (17)	0.037 (2)	0.0509 (19)	−0.0006 (15)	0.0157 (14)	−0.0048 (16)
C19	0.0418 (17)	0.0379 (19)	0.0440 (18)	−0.0030 (15)	0.0157 (14)	−0.0038 (15)
C20	0.0310 (16)	0.036 (2)	0.0445 (17)	0.0027 (14)	0.0093 (13)	−0.0075 (15)
C22	0.0324 (16)	0.033 (2)	0.0372 (17)	0.0017 (13)	0.0059 (13)	−0.0078 (14)

### Geometric parameters (Å, º)

**Table:**

O1—C1	1.377 (3)	C8—C9	1.389 (3)
O1—C9	1.383 (3)	C10—H10A	0.96
O2—C1	1.206 (3)	C10—H10B	0.96
N1—C11	1.344 (3)	C10—H10C	0.96
N1—C12	1.365 (3)	C11—C22	1.388 (3)
N2—C22	1.384 (3)	C12—C13	1.369 (4)
N2—C19	1.391 (3)	C12—H12	0.93
N2—H2	0.86	C13—C20	1.388 (4)
C1—C2	1.435 (4)	C13—H13	0.93
C2—C3	1.341 (3)	C14—C15	1.368 (4)
C2—H2A	0.93	C14—C18	1.392 (4)
C3—C8	1.447 (3)	C14—H14	0.93
C3—C11	1.494 (3)	C15—C16	1.388 (4)
C4—C5	1.374 (4)	C15—H15	0.93
C4—C8	1.388 (3)	C16—C17	1.385 (4)
C4—H4	0.93	C16—H16	0.93
C5—C6	1.394 (4)	C17—C19	1.384 (4)
C5—H5	0.93	C17—H17	0.93
C6—C7	1.379 (4)	C18—C19	1.396 (4)
C6—C10	1.510 (4)	C18—C20	1.445 (4)
C7—C9	1.378 (4)	C20—C22	1.405 (3)
C7—H7	0.93		
			
C1—O1—C9	121.7 (2)	H10A—C10—H10C	109.5
C11—N1—C12	117.9 (2)	H10B—C10—H10C	109.5
C22—N2—C19	108.0 (2)	N1—C11—C22	120.6 (2)
C22—N2—H2	126	N1—C11—C3	117.7 (2)
C19—N2—H2	126	C22—C11—C3	121.7 (2)
O2—C1—O1	117.0 (3)	C13—C12—N1	124.5 (3)
O2—C1—C2	126.4 (3)	C13—C12—H12	117.8
O1—C1—C2	116.6 (3)	N1—C12—H12	117.8
C3—C2—C1	123.3 (3)	C12—C13—C20	117.9 (3)
C3—C2—H2A	118.4	C12—C13—H13	121
C1—C2—H2A	118.4	C20—C13—H13	121
C2—C3—C8	119.0 (2)	C15—C14—C18	118.8 (3)
C2—C3—C11	119.7 (3)	C15—C14—H14	120.6
C8—C3—C11	121.3 (2)	C18—C14—H14	120.6
C5—C4—C8	121.1 (3)	C14—C15—C16	120.9 (3)
C5—C4—H4	119.4	C14—C15—H15	119.6
C8—C4—H4	119.4	C16—C15—H15	119.6
C4—C5—C6	121.0 (3)	C17—C16—C15	121.8 (3)
C4—C5—H5	119.5	C17—C16—H16	119.1
C6—C5—H5	119.5	C15—C16—H16	119.1
C7—C6—C5	118.6 (3)	C16—C17—C19	116.6 (3)
C7—C6—C10	120.3 (3)	C16—C17—H17	121.7
C5—C6—C10	121.0 (3)	C19—C17—H17	121.7
C6—C7—C9	119.7 (3)	C14—C18—C19	119.5 (3)
C6—C7—H7	120.1	C14—C18—C20	133.8 (3)
C9—C7—H7	120.1	C19—C18—C20	106.7 (2)
C9—C8—C4	117.0 (3)	C17—C19—N2	128.3 (3)
C9—C8—C3	118.0 (2)	C17—C19—C18	122.3 (3)
C4—C8—C3	124.9 (2)	N2—C19—C18	109.4 (2)
C7—C9—O1	116.1 (2)	C13—C20—C22	118.0 (3)
C7—C9—C8	122.5 (3)	C13—C20—C18	135.5 (3)
O1—C9—C8	121.4 (2)	C22—C20—C18	106.4 (2)
C6—C10—H10A	109.5	C11—C22—N2	129.7 (2)
C6—C10—H10B	109.5	C11—C22—C20	120.9 (3)
H10A—C10—H10B	109.5	N2—C22—C20	109.4 (2)
C6—C10—H10C	109.5		
			
C9—O1—C1—O2	179.9 (2)	C11—N1—C12—C13	−2.4 (4)
C9—O1—C1—C2	0.2 (4)	N1—C12—C13—C20	1.7 (4)
O2—C1—C2—C3	178.1 (3)	C18—C14—C15—C16	1.3 (5)
O1—C1—C2—C3	−2.2 (4)	C14—C15—C16—C17	−1.2 (5)
C1—C2—C3—C8	2.8 (4)	C15—C16—C17—C19	−0.2 (4)
C1—C2—C3—C11	−178.5 (3)	C15—C14—C18—C19	0.1 (4)
C8—C4—C5—C6	−1.1 (4)	C15—C14—C18—C20	178.5 (3)
C4—C5—C6—C7	1.8 (4)	C16—C17—C19—N2	−179.0 (3)
C4—C5—C6—C10	−177.1 (3)	C16—C17—C19—C18	1.6 (4)
C5—C6—C7—C9	−0.9 (4)	C22—N2—C19—C17	179.2 (3)
C10—C6—C7—C9	178.0 (3)	C22—N2—C19—C18	−1.3 (3)
C5—C4—C8—C9	−0.5 (4)	C14—C18—C19—C17	−1.6 (4)
C5—C4—C8—C3	−178.6 (3)	C20—C18—C19—C17	179.7 (3)
C2—C3—C8—C9	−1.4 (4)	C14—C18—C19—N2	178.9 (3)
C11—C3—C8—C9	179.8 (2)	C20—C18—C19—N2	0.1 (3)
C2—C3—C8—C4	176.6 (3)	C12—C13—C20—C22	0.7 (4)
C11—C3—C8—C4	−2.2 (4)	C12—C13—C20—C18	−179.2 (3)
C6—C7—C9—O1	179.3 (2)	C14—C18—C20—C13	2.6 (6)
C6—C7—C9—C8	−0.8 (4)	C19—C18—C20—C13	−178.9 (3)
C1—O1—C9—C7	−179.1 (2)	C14—C18—C20—C22	−177.4 (3)
C1—O1—C9—C8	1.0 (4)	C19—C18—C20—C22	1.1 (3)
C4—C8—C9—C7	1.5 (4)	N1—C11—C22—N2	−178.8 (2)
C3—C8—C9—C7	179.7 (2)	C3—C11—C22—N2	−0.5 (4)
C4—C8—C9—O1	−178.6 (2)	N1—C11—C22—C20	2.0 (4)
C3—C8—C9—O1	−0.4 (4)	C3—C11—C22—C20	−179.7 (2)
C12—N1—C11—C22	0.4 (4)	C19—N2—C22—C11	−177.3 (3)
C12—N1—C11—C3	−177.9 (2)	C19—N2—C22—C20	2.0 (3)
C2—C3—C11—N1	118.4 (3)	C13—C20—C22—C11	−2.5 (4)
C8—C3—C11—N1	−62.9 (3)	C18—C20—C22—C11	177.4 (2)
C2—C3—C11—C22	−60.0 (4)	C13—C20—C22—N2	178.1 (2)
C8—C3—C11—C22	118.8 (3)	C18—C20—C22—N2	−1.9 (3)

### Hydrogen-bond geometry (Å, º)

**Table:**

*D*—H···*A*	*D*—H	H···*A*	*D*···*A*	*D*—H···*A*
N2—H2···N1^i^	0.86	2.47	2.994 (3)	120

Symmetry code: (i) −*x*+1, *y*−1/2, −*z*+1/2.
